# Presentations and outcomes of familial hemophagocytic lymphohistiocytosis in the pediatric intensive care units (PICUs)

**DOI:** 10.3389/fped.2023.1152409

**Published:** 2023-04-18

**Authors:** Fahad Alsohime, Mohamad-Hani Temsah, Rawan M. Alotaibi, Reham M. Alhalabi, Sarah AlEnezy, Aly Abdelrahman Yousef, Abdullah Mohammed Alzaydi, Hussam Sameer Inany, Ayman Al-Eyadhy, Mohammed Almazyad, Ali Alharbi, Abdulaziz Abdullah Alsoqati, Abdurahman Andijani, Mohammed Abu Ghazal, Kamal El Masri, Maher Doussouki, Raheel Farooq Butt, Saleh Alshehri, Mohammed Alsatrawi, Jaramia Macarambon, Gamal M. Hasan, Abdulrahman Alsultan

**Affiliations:** ^1^ Department of Pediatrics, College of Medicine, King Saud University, Riyadh, Saudi Arabia; ^2^ Pediatric Intensive Care Unit, Department of Pediatrics, King Saud University Medical City, Riyadh, Saudi Arabia; ^3^ Prince Abdullah bin Khaled Coeliac Disease Research Chair, Department of Pediatrics, College of Medicine, King Saud University, Riyadh, Saudi Arabia; ^4^ College of Medicine, King Saud University Medical City, Riyadh, Saudi Arabia; ^5^ Division of Pediatric Critical Care, Department of Pediatrics, King Faisal Specialist Hospital and Research Center, Jeddah, Saudi Arabia; ^6^ Department of Pediatrics, Faculty of Medicine, Helwan University, Cairo, Egypt; ^7^ Pediatric Critical Care Division, Specialized Children Hospital, King Fahad Medical City, Riyadh, Saudi Arabia; ^8^ Pediatric Intensive Care Unit, King Fahad Specialist Hospital, Dammam, Saudi Arabia; ^9^ Pediatric Hematology & Oncology Department, King Fahad Specialist Hospital, Dammam, Saudi Arabia; ^10^ Pediatric Critical Care Division, King Saud Medical City, Riyadh, Saudi Arabia; ^11^ Pediatric Department, Assiut Faculty of Medicine, Assiut University, Assiut, Egypt; ^12^ Pediatric Department, Pediatric Intensive Care Unit, Sheikh Shakhbout Medical City, Abu Dhabi, United Arab Emirates; ^13^ Oncology Center, King Saud University Medical City, Riyadh, Saudi Arabia

**Keywords:** F-HLH: familial hemophagocytic lymphohistiocytosis, S-HLH: secondary hemophagocytic lymphohistiocytosis, hemophagocytic syndrome, pediatrics, intensive care, clinical features

## Abstract

**Objectives:**

We aimed to describe Familial Hemophagocytic Lymphohistiocytosis (F-HLH) patients' clinical features, intensive care courses, and outcomes.

**Methods:**

Multi-center retrospective cohort study of pediatric patients diagnosed with F-HLH from 2015 to 2020 in five tertiary centers in Saudi Arabia. Patients were classified as F-HLH based on their genetic confirmation of known mutation or on their clinical criteria, which include a constellation of abnormalities, early disease onset, recurrent HLH in the absence of other causes, or a family history of HLH.

**Results:**

Fifty-eight patients (28 male, 30 female), with a mean age of 21.0 ± 33.9 months, were included. The most common principal diagnosis was hematological or immune dysfunction (39.7%), followed by cardiovascular dysfunction in 13 (22.4%) patients. Fever was the most common clinical presentation in 27.6%, followed by convulsions (13.8%) and bleeding (13.8%). There were 20 patients (34.5%) who had splenomegaly, and more than 70% of patients had hyperferritinemia >500 mg/dl, hypertriglyceridemia >150 mg/dl and hemophagocytosis in bone marrow biopsy. Compared to deceased patients 18 (31%), survivors had significantly lower PT (*p* = 041), bilirubin level of <34.2 mmol/L (*p* = 0.042), higher serum triglyceride level (*p* = 0.036), and lesser bleeding within the initial 6 h of admission (*p* = 0.004). Risk factors for mortality included requirements of higher levels of hemodynamic (61.1% vs. 17.5%, *p* = 0.001) and respiratory (88.9% vs. 37.5%, *p* < 0.001) support, and positive fungal cultures (*p* = 0.046).

**Conclusions:**

Familial HLH still represents a challenge in the pediatric critical care setting. Earlier diagnosis and prompt initiation of appropriate treatment could improve F-HLH survival.

## Article summary

We describe Familial-HLH clinical features, PICU courses, and outcomes, as HLH still presents a pediatric challenge. Earlier diagnosis and prompt management could improve F-HLH survival.

## What's known on this subject

Familial hemophagocytic lymphohistiocytosis (F-HLH) and severe sepsis share similar inflammatory phenotypes, often leading to multiple organ dysfunction syndrome needing intensive care. Consequently, F-HLH is frequently mistaken for infection with severe sepsis, and its diagnosis is often delayed.

## What this study adds

This multi-center experience of F-HLH in PICUs over 5 years helps to identify the clinical course in the acute setting and recognize the clinical features that may be associated with worse outcomes, including mortality and ICU length of stay.

## Introduction

Hemophagocytic lymphohistiocytosis (HLH), also known as hemophagocytic syndrome, is a rare, often under-recognized life-threatening condition caused by uncontrolled activation of T lymphocytes and macrophages in addition to an impaired cytotoxic function of natural killer (NK) cells, which results in a massive cytokine release. As a result of failure to downregulate activated macrophages and lymphocytes, a hyperinflammatory phenotype ensues (1). The disease progresses rapidly in the absence of treatment with etoposide, immunosuppressive agents, or bone marrow (BM) transplantation (2).

The clinical presentation of HLH is variable. Cardinal signs and symptoms include fever, coagulopathy, hepatitis, CNS vasculitis and demyelination, acute respiratory distress syndrome, bone marrow hyperplasia or aplasia, pancytopenia, and hemophagocytosis (1). HLH commonly occurs in infancy, although it has been reported in all age groups. Over the last decade, the inclusion criteria of the HLH-2004 trial have been utilized for defining and diagnosing HLH (3). HLH is categorized as primary/familial (F-HLH; autosomal recessive) and secondary (S-HLH; triggered by infections, malignancy, and autoimmune disorders). Primary HLH is caused by genetic mutations of the FLH loci. Mutations in other genes (PRF1, UNC130, STX11, STBP2, RAB27A, LYST, SH2DIA, and XIAP) are usually associated with primary immunodeficiency syndrome have also been implicated in the pathogenesis. Secondary HLH occurs in the setting of bacterial and viral infections, malignancy (including T and B cell lymphomas), autoimmune diseases, and certain metabolic imbalances (4–9).

To our knowledge, the capacity to diagnose and treat HLH varies among different pediatric centers nationwide. We thus conducted a retrospective study on pediatric HLH in five tertiary hospitals in Saudi Arabia to investigate the epidemiologic, general clinical features, the PICU course, and the outcome of HLH.

## Materials and methods

We conducted a multicenter retrospective cohort study, assessing patients between the ages of 0–14 years, which is the age of the pediatric population/group in PICU in Saudi Arabia diagnosed with Familial HLH and admitted to PICU in one of five tertiary healthcare centers in three major cities in Saudi Arabia: King Khalid University Hospital (KKUH), King Fahad Medical City (KFMC) and King Saud Medical City (KSMC) in Riyadh, King Fahad Specialist Hospital (KFSH) in Dammam and King Faisal Specialist Hospital and Research Center (KFSHRC) in Jeddah from December 2019 to September 2020. The study protocol is approved by the institutional review boards of respective centers. HLH 2004 criteria (3) were implemented at the time of diagnosis. Data was obtained by reviewing electronic medical records and included family history, demographics, clinical and laboratory findings, therapeutic protocols, and Mortality within 28 days after PICU admission. All patients in our study were classified as F-HLH based on their genetic confirmation of known mutation or clinically suspected if a genetic diagnosis was not feasible, which include a clinical constellation of abnormalities, early disease onset, recurrent HLH in the absence of other causes, or family history of HLH.

The patient's system dysfunction was defined based on the Organ Dysfunction Criteria (10). Respiratory dysfunction was defined as PaO2/FIO2 less than 300 in the absence of cyanotic heart disease or preexisting lung disease, PaCO2 more than 65 torrs or 20 mm Hg over baseline, needing more than 50% FIO2 to maintain saturation ≥92%, or the need for non-elective invasive or noninvasive mechanical ventilation. Cardiac dysfunction was based on the presence of hypotension, need for vasoactive drugs to maintain BP, or two of the following: unexplained metabolic acidosis: base deficit, increased arterial lactate more than double the normal, oliguria, prolonged capillary refill, or core to peripheral temperature gap more than 3°C. Neurological dysfunction was defined as a Glasgow Coma Scale ≤11 or acute change in mental status with a decrease in GCS ≥3. Hepatic dysfunction was defined as total bilirubin ≥4 mg/dl or ALT 2 times the upper limit of normal for age. Renal dysfunction was defined as a serum creatinine ≥2 times the upper limit of normal for age or a 2-fold increase in baseline creatinine. Hematological dysfunction has been defined as cytopenia affecting at least 2 of lineages in peripheral blood: hemoglobin level <9 g/dl (anemia), total leucocytic count <4,000/µl (leucopenia), or platelet count <100,000/µl (thrombocytopenia) and coagulation disorder that was defined by a PT (prothrombin time) <50% and/or fibrinogen level <2 g/L or elevated fibrin degradation products (11–13). The immune dysfunction in HLH refers to the abnormality in the immune system's functioning, leading to the uncontrolled activation of T cells and macrophages. This activation results in the excessive production of cytokines such as interferon-gamma, tumor necrosis factor-alpha, and interleukin-6. The overproduction of cytokines leads to a hyperinflammatory state, causing tissue damage and the clinical features of HLH. Immune dysfunction was defined according to the HLH diagnostic criteria as follow: low or absent NK-cell activity and soluble CD25 (i.e., soluble IL-2 receptor) ≥2,400 U/ml (14).

We analyzed the data using the Statistical Package for Social Sciences (SPSS) version 23.0 (SPSS Inc, IBM, Armonk, New York, USA). Test of distribution normality was done using the Shapiro-Wilk test. Descriptive statistics are presented as numbers and percentages for categorical variables and as mean, standard deviation, or median and range for continuous variables based on variables' distribution. Significant difference in the proportions was done by the Chi-square test. The difference between means for normally distributed variables was made using the independent *t*-test, whereas the non-parametric Mann–Whitney *U* test was done for skewed distribution. *P-*values of <0.05 were considered statistically significant. The multivariate analysis was done to test for independent predictors for mortality (logistic regression) and prolonged PICU length of stay (linear regression). Variables that were deemed clinically meaningful or statistically significant in bivariate analyses were included in the regression models. The co-linearity among these factors was assessed using the variance inflation factor (VIF).

Ethical statement: Approval was granted by the institutional review board (IRB) of King Saud University, Riyadh, Saudi Arabia (Ref 19/0255/IRB, date December 26, 2019). The IRB waived the informed consent for the retrospective nature of data, and the study was conducted in accordance with the ethical standards of the IRB and with the Helsinki Declaration of 1975.

## Results

A total of 58 patients, 28 (48.3%) male, and 30 (51.7%) female, with a mean age of 21.0 ± 33.9 months, were included in the study. Fifty (86.2%) were Saudi nationals. First-degree consanguinity was seen in 22 (37.9%) patients. Table 1 shows the detailed demographic and clinical characteristics of all the patients.

**Table 1:** Demographic and clinical characteristics of studies patients diagnosed with F-HLH.

Variables	Number or Mean SD	%
Gender		
Male	28	48.3%
Female	30	51.7%
Male: female ratio	0.9–1%	
Nationality		
Saudi	50	86.2%
Non-Saudi	8	13.8%
Age		
Age in months	(Mean SD) 21.0 ± 33.9	Range 0–156
Age at diagnosis (Grouped)		
Less than 12 months	34	58.6%
More than 12 months	24	41.4%
Family History		
Positive Consanguinity	38	65.52%
Positive history for F-HLH	28	48.3%
Principal PICU diagnosis		
Hematology dysfunction	23	39.7%
Cardiovascular dysfunction	13	22.4%
Respiratory dysfunction	11	19.0%
Neuromuscular dysfunction	4	6.9%
Renal dysfunction	2	3.4%
Malignancy	1	1.7%
Metabolic dysfunction	1	1.7%
Not specified	3	5.2%
Clinical signs and symptoms at admission to PICU		
Splenomegaly	31	53.4%
Lymphadenopathy	6	10.3%
Convulsions (Total number)	8	13.8%
Focal convulsions	1	1.7%
Generalized convulsions	7	12.1%
Respiratory failure (Total)	53	91.38%
Type 1 respiratory failure	21	39.6%
Type 2 respiratory failure	6	11.3%
Mixed respiratory failure	2	3.8%
Not specified respiratory failure	24	45.3%
Bleeding tendency (Total)	8	13.8%
Needle pricks	1	1.7%
Petechial, bruises, epistaxis	1	1.7%
Pulmonary	4	6.9%
Pulmonary and Gastrointestinal	1	1.7%
Rectal	1	1.7%
Blood chemistry, cultures/PCR positivity		
Bacterial	13	22.4%
Fingal	6	10.3%
Viral	21	36.2%
Hyperferritinemia (>500 mg/dl)	41	70%
Hypertriglyceridemia (>150 mg/dl)	41	70%
Hemophagocytosis in the bone marrow	41	70%

The most common principal diagnosis was hematological dysfunction (*n* = 23, 39.7%) followed by cardiovascular dysfunction in 13 (22.4%) patients. Respiratory dysfunction was seen in 37 (63.8%), and hepatic dysfunction was noted in 29 patients (50.0%). Fever was the most common clinical presentation (*n* = 16, 27.6%), convulsions (*n* = 8, 13.8%), and bleeding (*n* = 8, 13.8%) four of which were pulmonary in nature**.** Based on the HLH-2004 diagnostic criteria and therapeutic guidelines for HLH, there were 31 (53.4%) who had splenomegaly >3 cm below the rib limit, and more than 70% of our patients had hyperferritinemia >500 mg/dl, hypertriglyceridemia >150 mg/dl and hemophagocytosis in the bone marrow (14).

**Figure 1:**
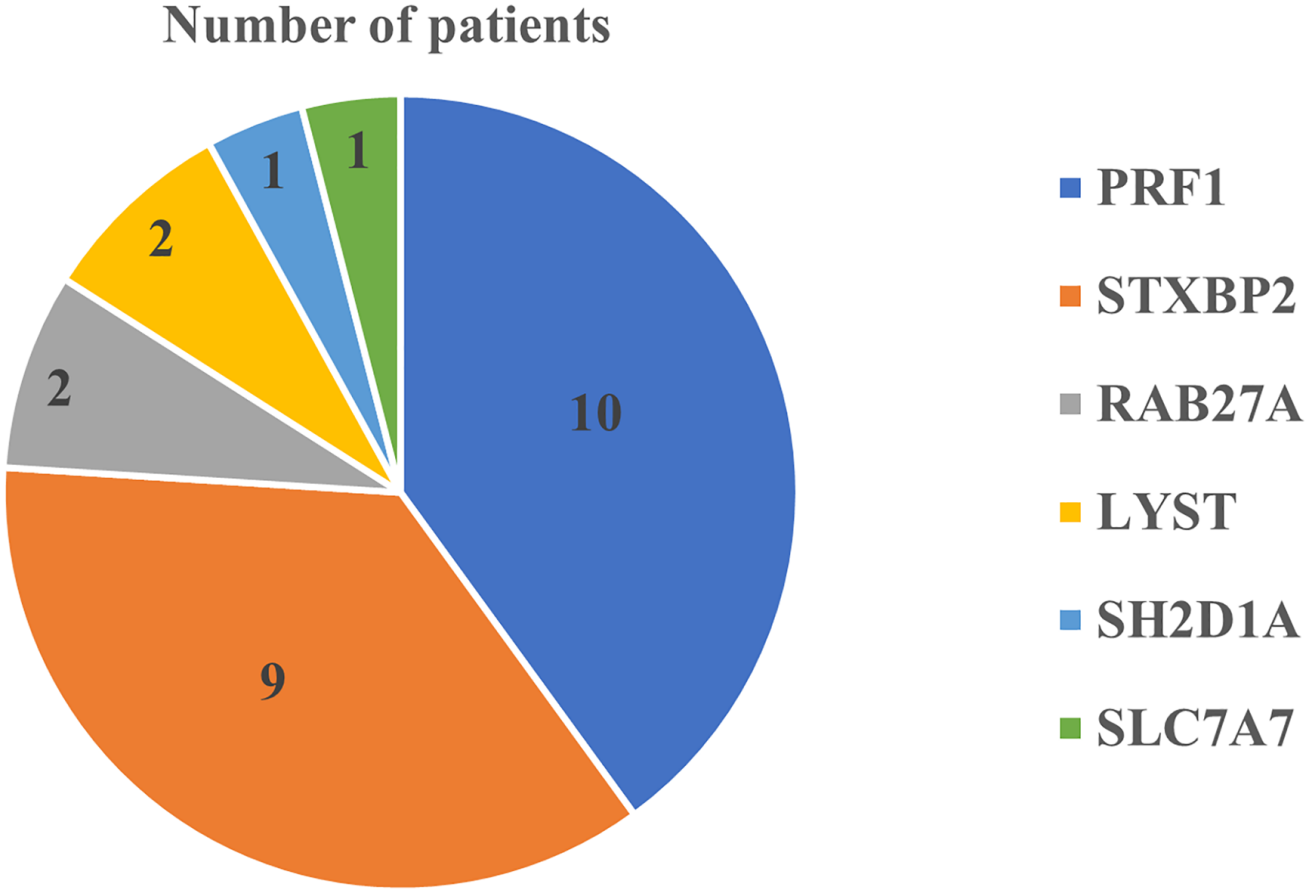
Identified F-HLH genes among studied patients.

Figure 1 shows the identified F-HLH genes among studied patient. We identified 25 patients (43%) with genetic diagnosis of F-HLH. The remaining patients (*n* = 33), we could not find a genetic testing for F-HLH at their medical records. They were diagnosed based on the diagnostic criteria of HLH as described in the methodology section.

Table 2 shows comparison of demographic and clinical characteristics among F-HLH survivors and non-survivors. Illness severity scores (PIM-2) showed significant differences between the two groups. Among the other variables, the use of invasive mechanical ventilation and the requirement of inotropes were significantly associated with non-survival, with *p*-values of <0.001 and 0.001, respectively. Bleeding observed within 6 h of PICU admission was also associated with mortality (*p*-value 0.004). Blood culture positive growth was seen in 40 patients (68.9%), positive for bacteria in 13 patients (22.4%), and fungi in 6 patients (10.3%), while 21 patients had positive results for viral PCR (36.2%). Fifteen bacterial cultures came up positive (two patients had two bacteria isolated), of which the most common bacterial isolate was *Staphylococcus epidermidis* in four cases, followed by *Klebsiella pneumoniae* in three cases, and two each for *Escherichia coli*, *Pseudomonas aeruginosa*, *Acinetobacter,* and *Stenotrophomonas maltophilia*. There was no significant difference in the proportion of patients who had positive cultures for bacteria or viruses as to survival (*p* = 0.981 and *p* = 0.370, respectively). However, a greater proportion of patients who had a positive culture for fungi did not survive (*p* = 0.046).

**Table 2:** Comparison of demographic and clinical characteristics between survivors and non-survivors.

Variables	All	Survivors Number = 40	Non-Survivors Number = 18	*p-*values
Demographic characteristics				
Age in months, Mean (SD)	21.0 (33.9)	23.2 (39.4)	16.2 ± 16.2	0.476
Age (<12 months), *n* (%)	34 (58.6%)	24 (60.0%)	10 (55.6%)	0.751
Male sex, *n* (%)	30 (51.7%)	21 (70.0%)	9 (30.0%)	0.860
Vital Signs				
Temperature at admission, Mean (SD)	36.9 (1.1)	36.9 (1.1)	37.0 ± 1.2	0.717
Heart rate, bpm, Mean (SD)	144.4 (29.2)	142.2 ± 29.2	149.0 ± 29.4	0.419
Respiratory rate per minute, Mean (SD)	43.2 (13.6)	42.6 ± 12.9	44.6 ± 15.1	0.621
Systolic BP, mmHg, Mean (SD)	96.8 (19.5)	97.6 ± 19.4	94.8 ± 20.1	0.617
Diastolic BP, mmHg, Mean (SD)	54.1 (16.6)	54.2 ± 15.6	53.9 ± 18.5	0.956
SPO2 at admission, Mean (SD)	96.4 (2.9)	96.6 ± 2.9	96.0 ± 2.8	0.440
Clinical Characteristics and Organs Dysfunction				
PIM-2 score, Median (IQR))	11.5 (15.4)	7.3 (10.2)	16.1 (18.7)	0.030
GCS at admission, Mean (SD)	12.1 (3.5)	12.1 ± 3.5	12.1 ± 3.4	0.993
CNS involvement, *n* (%)	18 (31.0%)	12 (30.0%)	6 (33.3%)	0.800
Cardiac dysfunction, *n* (%)	16 (28.1%)	9 (23.1%)	7 (38.9%)	0.217
Respiratory dysfunction, *n* (%)	37 (63.8%)	23 (57.5%)	14 (77.8%)	0.137
Hepatomegaly, *n* (%)	42 (72.4%)	30 (75.0%)	12 (66.7%)	0.511
Hepatic dysfunction, *n* (%)	29 (50.0%)	18 (45.0%)	11 (61.0%)	0.256
Splenomegaly, *n* (%)	31 (53.4%)	22 (56.4%)	9 (50.0%)	0.652
Lymphadenopathy, *n* (%)	6 (10.3%)	1 (5.6%)	5 (13.2%)	0.390
Bleeding within 6 h of admission, *n* (%)	8 (13.8%)	2 (5.0%)	6 (33.3%)	0.004
Positive blood cultures				
Positive culture for bacteria, *n* (%)	13 (22.4%)	9 (22.5%)	4 (22.2%)	0.981
Positive culture for fungi, *n* (%)	6 (10.3%)	2 (5.0%)	4 (22.2%)	0.046
Positive for viruses, *n* (%)	21 (36.2%)	16 (40.0%)	5 (27.8%)	0.370
Supportive treatment				
Low flow oxygen therapy, *n* (%)	20 (34.5%)	12 (60%)	8 (40%)	0.416
Low flow oxygen therapy days, mean (SD)	2.6 (2.1)	2.5 ± 2.0	3.0 ± 2.6	0.634
HFOT/ NIV, *n* (%)	18 (31.0%)	11 (27.5%)	7 (38.9%)	0.386
Conventional mechanical ventilation and HFOV, *n* (%)	31 (53.4%)	15 (37.5%)	16 (88.9%)	<0.001
Inotrope use, *n* (%)	18 (31.0%)	7 (17.5%)	11 (61.1%)	0.001
Inotrope use days, mean (SD)	3.8 (4.1)	4.0 ± 3.7	3.6 ± 4.8	0.878
CRRT use, *n* (%)	6 (10.3%)	3 (7.5%)	3 (16.7%)	0.289
CRRT use days,, mean (SD)	4.7 (3)	3.0 ± 2	6.3 ± 3	0.189
Antibiotic use, *n* (%)	53 (91.4%)	36 (90.0%)	17 (94.4%)	0.577
Antiviral use, *n* (%)	12 (20.7%)	8 (20.0%)	4 (22.2%)	0.847
Anticonvulsant use, *n* (%)	18 (31.0%)	12 (30.0%)	6 (33.3%)	0.800
HLH Treatment				
Initiation of therapy^a^	37 (63.8%)	25 (62.5%)	12 (66.7%)	0.760
HLH starting day since diagnosis or PICU admission (mean SD)	3.6 (2.1)	2.6 ± 3.4	1.9 ± 3.3	0.622
Steroids starting day since diagnosis or PICU admission (mean SD)	2.7 (3.5)	3.2 ± 3.6	1.6 ± 3.2	0.250
IVIG starting day since diagnosis or PICU admission (mean SD)	4.6 (2.4)	17 (42.5%)	6 (33.3%)	0.509
Anakinra, *n* (%)	2 (3.4%)	1 (2.5%)	1 (5.6%)	0.555
Outcome measures:				
PICU length of stay in days (mean SD)	12.5 (19.9)	9.0 ± 11.7	19.3 ± 28.9	0.075

N, number; SD, standard deviation;

PIM-2, pediatric index of mortality, GCS, glasgow coma scale; CNS, central nervous system; HFOT, high flow oxygen therapy; NIV, non-invasive ventilation; HVOF, high frequency oscillatory ventilation; CRRT, continuous renal replacement therapy; PICU, pediatric intensive care unit; IVIG, intravenous immunoglobulin.

^a^ Treatment of HLH according to 2004 guidelines recommendations.

Table 3 shows comparison of laboratory characteristics upon admission between survivors and non-survivors. Laboratory parameters showed that F-HLH patients who survived had significantly lower mean PT levels (*p* = 0.041). Mean serum triglyceride level was significantly higher among F-HLH patients who survived (*p* = 0.036). Mortality was higher among patients who had bilirubin levels of ≥34.2 mmol/L (*p* = 0.042), and significantly more patients who had bleeding within the 6 h of admission died (*p* = 0.004). Use of inotrope and invasive mechanical ventilation were significantly greater among patients who did not survive than those who survived (61.1% vs. 17.5%, *p* = 0.001 and 88.9% vs. 37.5%, *p* < 0.001, respectively). Positivity for fungal culture was significantly higher among patients who did not survive (22.2% vs. 5.0%, *p* = 0.046). No other significant differences in the proportion of patients and in the mean levels of parameters that were investigated between patients who survived and those who did not survive.

**Table 3:** Comparison of laboratory characteristics upon admission between survivors and non-survivors.

Variables	All	Normal range (Lab Results)	Survivors Number = 40	Non-Survivors Number = 18	*p-*values
Blood gas analysis					
PCO2, Mean (SD)	40.9 (19.3)	35–45 mmHg	42.4 ± 21.3	37.8 ± 14.1	0.411
PH, Mean (SD)	7.3 (0.2)	7.35–7.45	7.3 ± 0.2	7.3 ± 0.2	0.530
Hematological studies					
RBC, Mean (SD)	3.5 (1.8)	3.2–4.8 × 10^12^/L	3.6 ± 2.1	3.3 ± 0.9	0.530
Hb, Mean (SD)	8.7 (1.8)	9.5–13.5 gm/dL	8.6 ± 1.6	8.8 ± 2.2	0.747
WBC, median (IQR)	3.7 (6.7)	6–18 × 10^9^/L	4.8 (9.9)	2.2 (6.7)	0.475
Neutrophils, median (IQR)	1.9 (28.2)	1–9 × 10^9^/L	2.1 (23.3)	3.0 (67.1)	0.273
Lymphocytes, median (IQR)	16.8 (36.0)	2–17 × 10^9^/L	4.6 (45.5)	6.3 (29.2)	0.264
Eosinophils, median (IQR)	0 (0.1)	0.2–0.8 × 10^9^/L	0.01 (0.35)	0 (0.02)	0.359
Platelets, median (IQR)	45.0 (96.0)	140–450 × 10^9^/L	80.5 (136)	25.0 (77.0)	0.466
PT, median (IQR)	17.0 (8.1)	12–15 s	15.8 (5.6)	19.9 (10.5)	0.041
aPTT, median (IQR)	41.4 (22.8)	27–39 s	39.8 (23.6)	49.0 (51.2)	0.635
INR, median (IQR)	1.3 (1.1)	0.8–1.3 s	1.4 (0.5)	1.5 (2.7)	0.774
D-Dimer, median (IQR)	2.1 (9.1)	0.22 0.45 mcg/ml	1.3 (2.6)	7.2 (15.4)	0.018
Fibrinogen, Mean (SD)	1.6 (0.9)	2–4 gm/L	1.6 ± 1.0	1.4 ± 0.7	0.486
Blood chemistry and biochemical studies					
Blood urea, median (IQR)	3.8 (4.7)	1.4–6.8 mmol/L	4.0 (3.4)	4.4 (8.5)	0.371
Creatinine, median (IQR)	25.0 (30.5)	18–35 µmol/L	27.0 (18.7)	31.0 (42.5)	0.362
Uric acid, median (IQR)	160.0 (109.0)	120–320 µmol/L	127.0 (106.0)	189.0 (134.0)	0.656
Albumin, Mean (SD)	27.6 (5.7)	34–50 gm/L	27.8 ± 6.2	27.1 ± 4.6	0.685
Total proteins, Mean (SD)	50.4 (9.4)	56–75 gm/L	51.6 ± 10.4	47.9 ± 6.4	0.202
Ferritin, median (IQR)	4,390.0 (11,248.1)	13–150 mcg/L	4,390.0 (14,807.6)	5,836.5 (12,675.6)	0.453
Triglycerides, Mean (SD)	3.0 (1.7)	0.34–1.13 mmol/L	3.3 ± 1.7	2.3 ± 1.4	0.036
Cholesterol, Mean (SD)	3.2 (1.6)	3.2–5.2 mmol/L	3.2 ± 1.5	3.2 ± 2.0	0.958
Liver function tests					
Total bilirubin, median (IQR)	36.0 (92.4)	3–17 µmol/L	13.8 (45.2)	46.9 (68.0)	0.123
Direct bilirubin, median (IQR)	27.8 (90.1)	0–3 µmol/L	5.7 (38.2)	35.0 (65.0)	0.399
Indirect bilirubin, median (IQR)	9.8 (7.4)	2–17 µmol/L	8.4 (6.2)	11.0 (5.8)	0.745
AST, median (IQR)	342.7 (549.0)	15–37 unit/L	169.5 (488.0)	1,000.0 (2,690.0)	0.366
ALT, median (IQR)	123.0 (405.3)	20–65 unit/L	100.0 (144.0)	250.0 (865.0)	0.602
GGT, median (IQR)	439.0 (124.0)	5–55 unit/L	245.0 (174.0)	112.0 (225.0)	0.467
Serum immunoglobulins					
Ig E, median (IQR)	3.7 (9.2)	0.00–100 kU/L	7.1 (111.6)	3.7 (1.0)	0.701
Ig M, median (IQR)	3.8 (4.3)	0.200–0.870 gm/L	0.6 (0.6)	0.6 (0.7)	0.726
Ig G, median (IQR)	11.6 (5.6)	2.51–9.06 gm/L	7.6 (4.4)	7.0 (6.6)	0.166
Ig A, median (IQR)	2.9 (1.7)	0.013–0.530 gm/L	0.9 (0.9)	(0.8 (0.9)	0.920
Laboratory abnormalities (Grouped)					
Cytopenia, *n* (%)	37 (63.8%)	–	12 (66.7%)	25 (62.5%)	0.760
Neutrophil <1.0 × 10^9^/L, *n* (%)	17 (34.7%)	–	10 (28.6%)	7 (50.0%)	0.155
Hgb <90 g/L, *n* (%)	32 (55.2%)	–	23 (57.5%)	9 (50.0%)	0.595
Hemophagocytosis, *n* (%)	44 (75.9%)	–	28 (70.0%)	16 (88.9%)	0.120
Fibrinogen <1.5 g/L, *n* (%)	25 (50.0%)	–	17 (47.2%)	8 (57.1%)	0.529
Triglycerides ≥3 mmol/L, *n* (%)	18 (	–	15 (41.7%)	3 (20.0%)	0.140
Ferritin ≥500 ug/L, *n* (%)	46 (92.0%)	–	35 (92.1%)	11 (91.7%)	0.961
ALT ≥100 U/L, *n* (%)	26 (44.8%)	–	17 (42.5%)	9 (50.0%)	0.595
AST ≥100 U/L, *n* (%)	28 (62.2%)	–	18 (60.0%)	10 (66.7%)	0.664
Bilirubin ≥34.2 mmol/L, *n* (%)	28 (48.3%)	–	16 (41.0%)	12 (70.6%)	0.042

N, number; SD, standard deviation; IQR, inter-quartile range;

PT, prothrombin time; aPTT, activated partial thromboplastin time; INR, international neutralization ratio; AST, aspartate transaminase; ALT, alanine transaminase; GGT, gamma-glutamyl transpeptidase; Ig, immunoglobulin.

Table 4 shows the multivariate logistic regression analysis for independent predictor for mortality The PIM-2 scores and hepatic dysfunction were associated with mortality upon adjustment of the other factors.

**Table 4:** Multivariate logistic regression analysis for independent predictor for mortality.

	Coefficients						95% Confidence interval	
	Estimate	Standard Error	Standardized^a^	Odds Ratio	*z*	*p*	Lower bound	Upper bound
(Intercept)	2.555	0.891	1.707	12.869	2.868	0.004	0.809	4.301
Age (in months)	−0.001	0.007	−0.028	0.999	−0.092	0.927	−0.014	0.013
PIM-2 Score	−0.073	0.032	−1.106	0.930	−2.314	0.021	−0.135	−0.011
Hepatic Dysfunction, (Yes)	−1.991	0.774	−1.991	0.137	−2.574	0.010	−3.507	−0.475
Neurological Dysfunction, (Yes)	−0.986	0.774	−0.986	0.373	−1.274	0.203	−2.503	0.531
Renal Dysfunction, (Yes)	−2.439	1.300	−2.439	0.087	−1.876	0.061	−4.986	0.108

Survival (28 days’ survival of PICU admission) level “Yes” coded as class 1. Model performance metrics. AUC = 0.80, Sensitivity = 0.80, Specificity = 0.67, Precision = 0.75.

PIM-2, pediatric index of mortality-2.

^a^ Standardized estimates represent estimates where the continuous predictors are standardized (X-standardization).

Supplementary Table S1 shows the multivariate linear regression analysis for factors associated with prolonged PICU length of stay (LOS).

## Discussion

The present multicenter study is the first to report the clinical features and survival outcomes of children with F-HLH requiring PICU admission in Saudi Arabia. A total of 58 F-HLH patients were included in the study from four large PICUs.

The incidence of F-HLH in Saudi Arabia is unknown; however, it is expected to be higher than reports from North America and Europe, given the high consanguinity in our population (16–18). Thus, there is a need to develop a national protocol that standardizes the diagnostic and treatment approaches of children presenting with features of HLH and collect data prospectively, aiming to improve the survival of this life-threatening condition. The clinical team at Boston Children's Hospital published a very interesting collaborative approach to diagnosing and treating HLH and macrophage activation syndrome (MAS) and proposed quality improvement outcome measures such as time to HLH/MAS diagnosis, time to initiate therapy, length of hospital stay, need for ICU care, and mortality (19).

In our cohort, the most frequent organ dysfunction at PICU presentation was respiratory in two-thirds of patients, which seemed higher than what was reported in other HLH cohorts (20, 21). On the other hand, hepatic dysfunction in half of our patients, and neurological compromise in one-third were similar to other reports in the literature (20, 21). Nandhakumar et al., reported that among 52 children with HLH, 13% had Familial HLH, and 87% had secondary HLH. Their manifestations were fever in all of the 52 patients, organomegaly in 87%, respiratory distress in 54%, and neurological symptoms in 31% (22). Among another PICU HLH patients, mostly (*N* = 60/62) secondary HLH, had various complications, such as shock (71%), AKI (66.1%), ARDS (41.9%), DIC (54.8%), CNS dysfunction (54.8%), MODS (82.3%), and healthcare-associated infections (14.5%) (23). Therefore, Zhang et al. recommended from their literature review that, especially in the active familial HLH, surveillance should focus on potential complications, such as bleeding, respiratory distress, hypotension, neurologic involvement, malnutrition, liver, infection, or other organs failure (24).

It is noteworthy that the literature also compares the cytokine release syndrome which could lead to such multiorgan involvement in HLH with the COVID-19 cytokine storm (25). A case report of HLH associated with COVID-19 presents more clinical challenges that coinfection may cause in children (26). Future research on multimodal therapies for cytokine release syndrome could show more potential (27).

In our study, the most frequent causes of PICU admission are hematological derangement, followed by cardiovascular compromise and respiratory failure.

Nearly 20% of our cohort had an initial diagnosis of cardiovascular dysfunction (shock) upon PICU admission, and around 30% of the patients required vasoactive drugs during their PICU course (stay). A study by Li et al. has identified the shock as strong risk factor for mortality in pediatric patients with HLH (14), Whereas in another study by Parajuli et al. on 62 pediatric patients with HLH admitted to PICU, they reported that 70% of the patients had a primary diagnosis of shock and the non-survivors had a higher need for vasoactive drugs (23).This association was demonstrated in another case series by Leow et al. (28). Our analysis's result was consistent with these reports. where we found that the non-survivors had a higher need for inotropes (28).

Coagulopathy and bleeding among HLH patients represent a major challenge in the critical care setting that necessitate a proper investigation and therapeutic intervention to control it. In our cohort, eight patients had significant bleeding within 6 h of admission to the PICU, half of them, the source of bleeding was pulmonary hemorrhage. Our analysis showed that the bleeding was significantly associated with non-survivors. There was no significant statistical difference between the survivors and non-survivors in terms of hematological laboratory results such as hemoglobin level, WBC, and platelets count, and APTT, except for PT which was significantly shorter in the survivor group. Several reports indicated that the need of high blood product transfusion and coagulopathy were factors associated with higher mortality in children with HLH (29, 30).

Our study showed that the overall mortality rate during the PICU stay was 31%. The mortality rate ranged between 21% and 59% in a few studies that assessed the PICU outcome of children with HLH (23, 28, 31–33), HLH-94 and HLH-2004 studies did not report the outcome of HLH patients requiring ICU care; however, the mortality rate at two months was 14% in both studies (34, 35).

Thus, it is essential to optimize PICU care to lower the rate of early morbidities and mortality, aiming to further improve the outcome of F-HLH as the probability of survival remains suboptimal at 5-year and ranges between 51% and 59%, as reported in HLH-94, HLH-2004, and a large Saudi single-institution experience (34–36). The multivariate analysis for prolonged PICU LOS revealed that younger age, higher PIM-2 scores, direct bilirubin, fibrinogen, creatinine, and lower triglycerides and platelets. This multisystem involvement reflects the level of organ support and the need for critical care-related monitoring and therapeutics. Another interesting observation is that 41% enrolled in this study had an unscheduled PICU readmission. This is significantly higher than the reported 2.5%–3.7% rate of unscheduled PICU readmission among children with various disorders who requires PICU (37–39). It is crucial to identify factors contributing to the high PICU readmission in our population, given that inferior outcome was reported in children requiring PICU readmission (37, 38, 40).

Factors associated with increased risk of mortality in our study included the need for mechanical ventilation, inotropes use, hyper-bilirubinemia, prolonged prothrombin time, bleeding within 6 h of PICU admission, and fungal infections. There was a statistically significant higher median PIM scores among non-survivors compared to survivors, as expected. Compared to neurological and renal dysfunction, the involvement of hepatic dysfunction and its association with mortality in bivariate analysis was consistent with multivariate analysis when adjusted for age and critical illness severity score. Most of our patients were transferred from the inpatient floor to PICU (58%) with relatively long hospital admission prior to the transfer, an average of 9.9 days. It is unclear whether a delay in reaching the diagnosis of HLH or initiating proper therapy and supportive care contributed to the need for ICU care in our study (41).

In other studies, among HLH patients requiring PICU admission, poor prognostic factors included a worse PIM score, mechanical ventilation, dialysis, frequency of blood transfusion, and inotropic support (23, 28). Risk factors of early mortality in children with newly diagnosed HLH, regardless of the need for PICU care, included hyperbilirubinemia, high ferritin >2,000 µg⁄L, CSF pleocytosis, respiratory failure, coagulopathy, MODS, neutropenia, and hypoalbuminemia (14, 30, 42). Most of these factors are not modifiable, highlighting the need for prompts diagnosis of HLH and timely initiation of HLH therapy to prevent disease progression and the need for ICU care.

Interestingly, our study showed that the proportion of patients on HLH-2004 and the starting days of this therapy was comparable between survivors and non-survivors. HLH-2004 treatment and steroids were started early in our patients, an average of 3 days from PICU admission. This probably indicates that pre-PICU management had an influence on the outcome in our cohort.

The results of this study need to be interpreted in view of its limitations. Given the low prevalence and rare occurrence of the F-HLH, a relatively small number of cases were included despite the multicenter approach. In addition, the data represent the PICU cohort of F-HLH, which needs to be considered for external validation when comparing other non-PICU cohorts of F-HLH. For instance, we did not collect data on the timing of the diagnosis of HLH and treatment before the PICU transfer.

## Conclusion

Among our studied patients with F-HLH, survivors had significantly lower PT, lower bilirubin level and lesser bleeding within the initial 6 h of admission. Contrary, risk factors for mortality included higher levels of hemodynamic and respiratory support, and positive fungal cultures. The current study emphasizes the need to have a better characterization of F-HLH in the PICU settings, which represents diagnostic and therapeutic challenges and relatively exhaust the resources for multi-organ support. F-HLH still represents a challenge in the pediatric critical care setting, and earlier diagnosis and prompt management could improve survival.

## Data Availability

The original contributions presented in the study are included in the article/**Supplementary Material**, further inquiries can be directed to the corresponding author.

## References

[1] Filipovich A McClain K Grom A. Histiocytic disorders: recent insights into pathophysiology and practical guidelines. Biol Blood Marrow Transplant. (2010) 16:S82–9. 10.1016/j.bbmt.2009.11.014 19932759

[2] Blanche S Caniglia M Girault D Landman J Griscelli C Fischer A. Treatment of hemophagocytic lymphohistiocytosis with chemotherapy and bone marrow transplantation: a single-center study of 22 cases. Blood. (1991) 78:51–4. 10.1182/blood.V78.1.51.51 2070059

[3] Henter JI Horne A Aricó M Egeler RM Filipovich AH Imashuku S HLH-2004: diagnostic and therapeutic guidelines for hemophagocytic lymphohistiocytosis. Pediatr Blood Cancer. (2007) (48):124–31. 10.1002/pbc.21039 16937360

[4] Janka G Imashuku S Elinder G Schneider M Henter JI. Infection- and malignancy-associated hemophagocytic syndromes: secondary hemophagocytic lymphohistiocytosis. Hematol Oncol Clin North Am. (1998) 12:435–44. 10.1016/S0889-8588(05)70521-9 9561911

[5] Rosado FGN. Kim as: hemophagocytic lymphohistiocytosis: an update on diagnosis and pathogenesis. Am J Clin Pathol. (2013) 139:713–27. 10.1309/AJCP4ZDKJ4ICOUAT 23690113

[6] Mou SS Nakagawa TA Riemer EC McLean TW Hines MH Shetty AK. Hemophagocytic lymphohistiocytosis complicating influenza A infection. Pediatrics. (2006) 118:e216–9. 10.1542/peds.2005-1861 16785288

[7] Yuzurihara SS Ao K Hara T Tanaka F Mori M Kikuchi N Human parechovirus-3 infection in nine neonates and infants presenting symptoms of hemophagocytic lymphohistiocytosis. J Infect Chemother. (2013) 19:144–8. 10.1007/s10156-012-0420-9 22569793

[8] Usmani GN Woda BA Newburger PE. Advances in understanding the pathogenesis of HLH. Br J Haematol. (2013) 161:609–22. 10.1111/bjh.12293 23577835

[9] Gholam C Grigoriadou S Gilmour KC Gaspar HB. Familial haemophagocytic lymphohistiocytosis: advances in the genetic basis, diagnosis and management. Clin Exp Immunol. (2011) 163:271. 10.1111/j.1365-2249.2010.04302.x 21303357 PMC3048610

[10] Goldstein B Giroir B Randolph A. International pediatric sepsis consensus conference: definitions for sepsis and organ dysfunction in pediatrics. Pediatr Crit Care Med. (2005) 6:2–8. 10.1097/01.PCC.0000149131.72248.E6 15636651

[11] Kumar R Kalra SP Kumar H Anand AC Madan M. Pancytopenia-A six year study. J Assoc Physicians India. (2001) 49:1079-81. PMID: 11868860

[12] Henter JI Elinder G Os TA. The FHL study group of the histiocyte society. Diagnostic guidelines for hemophagocytic lymphohistiocytosis. Semin Oncol. (1991) 18:29-33. PMID: 1992521

[13] Valade S Azoulay E Galicier L Boutboul D Zafrani L Stepanian A Coagulation disorders and bleedings in critically ill patients with hemophagocytic lymphohistiocytosis. Medicine. (2015) 94(40). PMID: . PMCID: PMC4616770. 10.1097/MD.0000000000001692 26448017 PMC4616770

[14] Henter JI Horne AC Arico M Egeler M Alexandra H Filipovich AH HLH-2004: Diagnostic and therapeutic guidelines for hemophagocytic lymphohistiocytosis. Pediatr Blood Cancer (2007) 48:124–31. PMID: . 10.1002/pbc.21039 16937360

[15] Li X Yan H Zhang X Huang J Xiang ST Yao Z Clinical profiles and risk factors of 7-day and 30-day mortality among 160 pediatric patients with hemophagocytic lymphohistiocytosis. Orphanet J Rare Dis. (2020) 15:1–11. 10.1186/s13023-019-1279-y 32867836 PMC7456759

[16] El-Hazmi MAF Al-Swailem AR Warsy AS Al-Swailem AM Sulaimani R Al-Meshari AA. Consanguinity among the Saudi Arabian population. J Med Genet. (1995) 32:623–6. 10.1136/jmg.32.8.623 7473654 PMC1051637

[17] Henter JI Elinder G Söder O Ost A. Incidence in Sweden and clinical features of familial hemophagocytic lymphohistiocytosis. Acta Paediatr Scand. (1991) 80:428–35. 10.1111/j.1651-2227.1991.tb11878.x 2058392

[18] Allen CE McClain KL. Pathophysiology and epidemiology of hemophagocytic lymphohistiocytosis. Hematology. (2015) 2015:177–82. 10.1182/asheducation-2015.1.177 26637718

[19] Halyabar O Chang MH Schoettler ML Schwartz MA Baris EH Benson LA Calm in the midst of cytokine storm: a collaborative approach to the diagnosis and treatment of hemophagocytic lymphohistiocytosis and macrophage activation syndrome. Pediatr Rheumatol. (2019) 17:1–12. 10.1186/s12969-019-0309-6 PMC6376762 30764840

[20] Henter JI Nennesmo I. Neuropathologic findings and neurologic symptoms in twenty-three children with hemophagocytic lymphohistiocytosis. J Pediatr. (1997) 130:358–65. 10.1016/S0022-3476(97)70196-3 9063409

[21] Kerguenec C Hillaire S Molinié V Gardin C Degott C Erlinger S Valla D. Hepatic manifestations of hemophagocytic syndrome: a study of 30 case. Am J Gastroenterol. (2001) 96:852–7. 10.1016/S0002-9270(00)02425-4 11280564

[22] Nandhakumar D Loganatha A Sivasankaran M Sivabalan S Munirathnam D. Hemophagocytic lymphohistiocytosis in children. Indian J Pediatr. (2020) 87:526–31. 10.1007/s12098-020-03190-6 32056194

[23] Parajuli B Angurana SK Awasthi P Nallasamy K Baranwal A Bansal A Hemophagocytic lymphohistiocytosis in a PICU of a developing economy: clinical profile, intensive care needs, outcome, and predictors of mortality. Pediatr Crit Care Med. (2021) 22:E44–57. 10.1097/PCC.0000000000002539 33031348

[24] Adam MP Mirzaa GM Pagon RA Genereviews®. Seattle: University of Washington (2022).

[25] Kim JS Lee JY Yang JW Lee KH Effenberger M Szpirt W Immunopathogenesis and treatment of cytokine storm in COVID-19. Theranostics. (2021) 11:316–29. 10.7150/thno.49713 33391477 PMC7681075

[26] Jose PMM Paola ZS Eduardo DG Osvaldo Arturo SMM Fernando BG. A case of coinfection of a pediatric patient with acute SARS-COV-2 with MIS-C and severe DENV-2 in Mexico: a case report. BMC Infect Dis. (2021) 21:1–7. 10.1186/s12879-020-05706-z 34663252 PMC8521498

[27] Bottari G Merli P Guzzo I Stoppa F Ruggeri A Nardo MD Multimodal therapeutic approach of cytokine release syndrome developing in a child given chimeric antigen receptor-modified T cell infusion. Crit Care Explor. (2020) 2:e0071. 10.1097/CCE.0000000000000071 32166291 PMC7063902

[28] Leow EH Soh SY Tan AM Mok YH Chan MY Lee JH. Critically ill children with hemophagocytic lymphohistiocytosis: a case series of 14 patients. J Pediatr Hematol Oncol. (2017) 39:e303–6. 10.1097/MPH.0000000000000916 28697170

[29] Dao ATT Luong VT Nguyen TT Huynh QTV Phan TTT Lam MT Risk factors for early fatal outcomes among children with hemophagocytic lymphohistiocytosis (HLH): a single-institution case-series in Vietnam. Pediatr Hematol Oncol. (2014) 31:271–81. 10.3109/08880018.2013.858198 24308730

[30] Trottestam H Berglöf E Horne A Onelöv E Beutel K Lehmberg K Risk factors for early death in children with haemophagocytic lymphohistiocytosis. Acta Paediatr. (2012) 101:313–8. 10.1111/j.1651-2227.2011.02501.x 22017632

[31] Karapinar B Yilmaz D Balkan C Akin M Ay Y Kvakli K. An unusual cause of multiple organ dysfunction syndrome in the pediatric intensive care unit: hemophagocytic lymphohistiocytosis. Pediatr Crit Care Med. (2009) 10:285–90. 10.1097/PCC.0b013e318198868b 19433941

[32] Gregory J Greenberg J Basu S. Outcomes analysis of children diagnosed with hemophagocytic lymphohistiocytosis in the PICU. Pediatr Crit Care Med. (2019) 20:E185–90. 10.1097/PCC.0000000000001827 30520798

[33] Dao D Xoay TD Galeano BK Phuc PH Ouellette Y. Etiologies and clinical outcomes of patients with secondary hemophagocytic lymphohistiocytosis at a tertiary PICU. Pediatr Crit Care Med. (2019) 20:e311–8. 10.1097/PCC.0000000000001980 31149968

[34] Henter JI Samuelsson-Horne A Aricò M Egeler RM Elinder G Filipovich AH Treatment of hemophagocytic lymphohistiocytosis with HLH-94 immunochemotherapy and bone marrow transplantation. Blood. (2002) 100:2367–73. 10.1182/blood-2002-01-0172 12239144

[35] Bergsten E Horne A Aricó M Astigarraga I Egeler RM Filipovich AH Confirmed efficacy of etoposide and dexamethasone in HLH treatment: long-term results of the cooperative HLH-2004 study. Blood. (2017) 130:2728–38. 10.1182/blood-2017-06-788349 28935695 PMC5785801

[36] Al Ahmari A Alsmadi O Sheereen A Elamin T Jabr A El-Baik L Genetic and clinical characteristics of pediatric patients with familial hemophagocytic lymphohistiocytosis. Blood Res. (2021) 56:86–101. 10.5045/br.2021.2020308 34083498 PMC8246041

[37] Czaja AS Hosokawa PW Henderson WG. Unscheduled readmissions to the PICU: epidemiology, risk factors, and variation among centers. Pediatr Crit Care Med. (2013) 14:571–9. 10.1097/PCC.0b013e3182917a68 23823192

[38] Mat Bah MN Syed Mohamed SA Abdullah N Yusnita Alias E. Unplanned PICU readmission in a middle-income country: who is at risk and what is the outcome? Pediatr Crit Care Med. (2020) 21:E959–66. 10.1097/PCC.0000000000002406 32590834

[39] Kotsakis A Stevens D Frndova H Neal R Williamson G Mohseni-Bod H Description of PICU unplanned readmission. Pediatr Crit Care Med. (2016) 17:558–62. 10.1097/PCC.0000000000000735 27261644

[40] Kalzén H Larsson B Eksborg S Lindberg L Edberg KE Frostell C. Survival after PICU admission: the impact of multiple admissions and complex chronic conditions. PLoS One. (2018) 13:e0193294. 10.1371/journal.pone.0193294 29621235 PMC5886395

[41] Li X Yan H Xiao Z Zhang X Huang J Xiang ST Diagnostic time lag of pediatric haemophagocytic lymphohistiocytosis and patient characteristics: a retrospective cohort study. Front Pediatr. (2021) 9:1–12. 10.3389/fped.2021.692849 PMC8247774 34222154

[42] Bin Q Gao JH Luo JM. Prognostic factors of early outcome in pediatric hemophagocytic lymphohistiocytosis: an analysis of 116 cases. Ann Hematol. (2016) 95:1411–8. 10.1007/s00277-016-2727-6 27307280

