# Supplementary material for: Presentations and outcomes of familial hemophagocytic lymphohistiocytosis in the pediatric intensive care units (PICUs)
Source: Front Pediatr. 2023 Apr 18;11:1152409. doi: 10.3389/fped.2023.1152409 (PMC10151775; doi:10.3389/fped.2023.1152409)
Supplement: Supplementary file 1 [file Table1.docx]

**Presentations and Outcomes of Familial Hemophagocytic Lymphohistiocytosis in the Pediatric Intensive Care Units ICUs (PICUs)**

**Supplemental Digital Content**

**Supplementary Table 1:** Multivariate linear regression analysis***** for factors associated with prolonged pediatric intensive care unit (PICU) length of stay (LOS)

**Supplementary Table 1:** Multivariate linear regression analysis***** for factors associated with prolonged pediatric intensive care unit (PICU) length of stay (LOS)

| **Coefficients** | | | | | | | | **95% CI** | | |
| --- | --- | --- | --- | --- | --- | --- | --- | --- | --- | --- |
| **Variable** | **Unstandardized** | **Standard Error** | **Standardized** | | **t** | | **p** | **Lower** | **Upper** | |
| (Intercept) | 66.149 | 20.050 |  | 3.299 | | 0.009 | | 20.794 | | 111.505 |
| Age in months | -0.151 | 0.065 | -0.364 | -2.339 | | **0.044** | | -0.297 | | -0.005 |
| PIM-2 Score | 1.062 | 0.281 | 0.566 | 3.785 | | **0.004** | | 0.427 | | 1.697 |
| Initial BP | -0.288 | 0.236 | -0.271 | -1.223 | | 0.252 | | -0.821 | | 0.245 |
| Neutrophil count | 0.160 | 0.130 | 0.233 | 1.230 | | 0.250 | | -0.134 | | 0.453 |
| Fibri0gen | 19.573 | 6.458 | 0.711 | 3.031 | | **0.014** | | 4.963 | | 34.183 |
| Creatinine | 0.379 | 0.135 | 0.462 | 2.805 | | **0.021** | | 0.073 | | 0.685 |
| Ferritin | -2.090e -4 | 2.361e -4 | -0.133 | -0.885 | | 0.399 | | -7.431e -4 | | 3.252e -4 |
| Triglycerides | -9.873 | 3.753 | -0.553 | -2.630 | | **0.027** | | -18.363 | | -1.382 |
| Direct Bilirubin | -0.209 | 0.087 | -0.369 | -2.394 | | **0.040** | | -0.406 | | -0.011 |
| ALT | 0.008 | 0.009 | 0.160 | 0.941 | | 0.371 | | -0.011 | | 0.028 |
| Platelets | -0.250 | 0.050 | -1.425 | -5.010 | | **< .001** | | -0.363 | | -0.137 |

*The model was performed using ANOVA with R² =0.885

**PIM-2:** Pediatric Index of Mortality-2 score; **BP:** Blood Pressure; **ALT:** Alanine Transaminase;
